# Experimental Analysis of Stress Shielding Effects in Screw Spacers Placed in Porcine Spinal Tissue

**DOI:** 10.3390/jfb15080238

**Published:** 2024-08-22

**Authors:** Elliot Alonso Alcántara-Arreola, Karla Nayeli Silva-Garcés, Jocabed Mendoza-Martínez, Miguel Antonio Cardoso-Palomares, Christopher René Torres-SanMiguel

**Affiliations:** Instituto Politécnico Nacional, Escuela Superior de Ingeniería Mecánica y Eléctrica, Unidad Zacatenco, Sección de Estudios de Posgrado e Investigación, Ciudad de México 07738, Mexicojmendozam1903@alumno.ipn.mx (J.M.-M.);

**Keywords:** biomechanics, lumbar interbody fusion, experimental test, endoprosthesis, stress shielding

## Abstract

Bone cortical tissues reorganize and remodel in response to tensile forces acting on them, while compressive forces cause atrophy. However, implants support most of the payload. Bones do not regenerate, and stress shielding occurs. The aim is to analyze the biomechanical behavior of a lumbar cage to study the implant’s stress shielding. The ASTM E-9 standard was used with the necessary adjustments to perform compression tests on lumbar and thoracic porcine spinal vertebrae. Twelve cases were analyzed: six with the metal prosthesis and six with the PEEK implant. A mathematical model based on the Hertz contact theory is proposed to assess the stress shielding for endoprosthesis used in spine pathologies. The lumbar spacer (screw) helps to reduce the stress shielding effect due to the ACME thread. The best interspinous spacer is the PEEK screw. It does not embed in bone. The deformation capability increases by 11.5% and supports 78.6 kg more than a system without any interspinous spacer.

## 1. Introduction

Spinal degenerative disease (SDD) refers to a collection of medical conditions characterized by persistent discomfort in the neck, upper back, or lower back [1]. Spinal injuries and diseases of the spinal column are becoming more prevalent in contemporary society [2]. Back pain is a global issue and has been a leading factor in causing disability over the past three decades [3]. This can be attributed to the rising prevalence of overweight, obesity, and diminished muscular strength linked to SDD in individuals of all genders and age groups [4]. The primary factor responsible for low back discomfort is typically the degeneration of the intervertebral discs [5]. Currently, numerous conventional non-surgical approaches exist to address these diseases. The medical field prioritizes the prevention, significance, and treatment of SDD through physical activation (stretching exercises and yoga) and health-focused education (ergonomics, self-management skills, pain neuroscience education, and stress reduction approaches) [6]. The last alternative to relieve the pain caused by SDD is surgery.

In recent decades, there has been a growing interest in the use of the intervertebral fusion technique to treat degenerative spinal disorders in patients who do not show improvement with other methods [7,8,9,10,11]. Lumbar fusion is accomplished with the use of spine implants (SIs). A device is designed to support the bone implants and preserve the natural height and curve of the spinal space following surgery. SDD can be efficiently treated by implementing an SI. The impact of surgery is mostly determined by the biomechanical characteristics of orthopedic implants, which are influenced by their size, shape, and composition [12].

Bone’s mechanical properties are also important. A healthy bone of a person or animal remodels itself depending on the loads to which it is subjected [13,14]. Hard tissue must support a large amount of load to remain healthy. The SI must be placed in a damaged section to heal so that it does not allow the bone to support any load in the fractured area. Because of this, bone loses density. This phenomenon is referred to as stress shielding (*SS*) [15,16,17,18,19,20,21,22]. This effect is problematic if the difference between the stiffness of the bone and the implant is considerable. As an example, the elastic modulus of cortical bone ranges from 1 to 20 GPa, whereas the elastic modulus of trabecular bone ranges from 0.02 to 6 GPa. In comparison, the elastic modulus of titanium is 110 GPa. As a result of the notable variation in elastic modulus, the implant does not transfer loads to the bone tissue, leading to *SS*. This results in a reduction in the mechanical rigidity of the bone [23]. Several researchers have conducted studies on *SS*. Meena et al. [2] specifically focused on investigating the stresses and deformations in porous SI under different loading conditions using the finite element method (FEM). The results indicated that the reduction in *SS* was primarily attributed to the pore size, which ranged from 0.4 to 0.6 mm. It can be concluded that the impact of pore size on the implant structure is more significant.

Tsuang et al. [24] designed a biomimetic porous SI. They performed simulations and mechanical tests to study stresses and deformations. They adhered to the manufacturing standards set by the American Society for Testing and Materials International (ASTM) in order to enhance the design process and minimize expenses and material usage. They concluded that the heat treatment applied to the SI improved the apparent properties of the material, optimizing the stiffness of the device. This reduces the *SS* and, at the same time, provides adequate space for bone growth. Harikrishna et al. [25] studied the influence of pedicle screw dimensions after implant placement and evaluated the device displacement and stress using the FEM. The study emphasizes the influence of screw diameter on the displacement and stresses exerted on the bone throughout various postoperative stages. It was noted that a thread with a width of 0.5 mm exhibits enhanced stress transmission, resulting in decreased *SS* and enhanced bone remodeling.

Safavi et al. [26] conducted a systematic review in which they analyzed 46 articles focusing on studies of hip, knee, and shoulder prostheses. It was determined that several investigations did not utilize quantitative methods to depict benefits after surgery. The primary factors (such as materials, porosity, and geometry) that decrease the *SS* vary among different research. Nevertheless, considering the characteristics of the methodologies used, the examined designs had the highest potential. While porous designs typically decrease *SS*, additional research and clinical trials are necessary to establish the most effective design approach for prosthetic implants. Yan et al. [17] proposed an SI that is a porous intervertebral cage optimized through topology. FEM was used to perform SI biomechanical analysis comparing titanium and a PEEK case. The results showed that the SI they proposed has a smaller stress zone between the bone and the cage. The SI has higher stresses in the bone and strain energy deformation compared to the other two cases, demonstrating less embedding of the prosthesis into the bone and a lower risk of *SS*. It has been shown that in most of the reported literature cases, the study of the *SS* is carried out by FEM. Implant biomechanical analysis by experimental tests is critical to ensure that implants will operate correctly once they are placed in the patient. O. Ramirez et al. [27] conducted an experimental assessment to investigate the mechanical characteristics of both the rib and the plate in the design of an osteosynthesis implant for rib fractures. Simultaneously, they subjected them to compression cycles that mimic the respiratory function carried out by humans. The researchers determined that the implant’s design is sufficient and does not require any alterations to enable its use in medical contexts.

Another variable to consider that can impact spinal stability is the thickness of the cortical bone in the vertebrae. K. Magalhães et al. [28] took measurements of cortical bone thickness and bone mineral density (BMD) at the L2 vertebra for comparison with the corresponding measurements at the L3 and L4 vertebrae. The researchers utilized computed tomography (CT) to quantify the thickness of the cortical bone at the L2 vertebra. Additionally, bone densitometry (DEXA) scans were employed for BMD. The study findings suggest a clear correlation between reduced cortical bone thickness and decreased BMD in individuals diagnosed with osteopenia and osteoporosis. The study findings emphasize that there is significant variation in BMD and cortical bone thickness across different skeletal areas and vertebrae.

The work aims to perform experimental compression tests on vertebrae with different SIs to analyze the biomechanical behavior and the embedding process of the SI in the bone through the *SS*.

## 2. Materials and Methods

**Figure 1:**
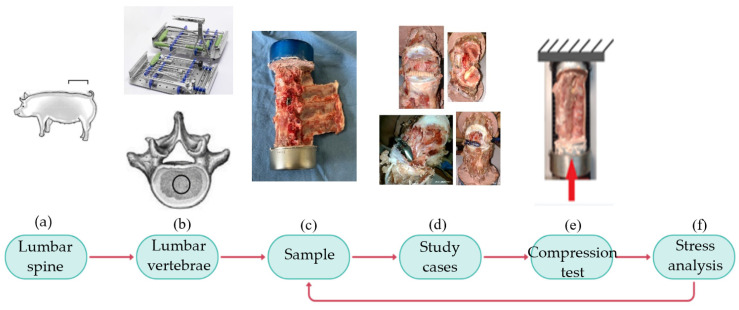
Methodology. (**a**) Location of the lumbar region to remove the vertebrae. (**b**) Lumbar vertebrae and medical instrumentation. (**c**) Sample fixation to cans with a dental cast. (**d**) Titanium and PEEK screws; titanium and PEEK cages. (**e**) Compression test diagram. (**f**) Evaluation of the obtained results.

Figure 1 summarizes the methodology. First (Figure 1a), vertebrae L2–L5 are removed from the porcine lumbar region. Figure 1b shows the medical instrumentation (pedicle probe, pedicle awl, pedicle screwdriver, rod bender, rongeurs, drill guides, and surgical retractors) to drill a hole in the soft tissue. The drill hole should be 2 mm smaller than the implant dimensions to ensure proper tightening. Afterward, a sample is fixed into cans with a dental cast (Figure 1c). The experimental design evaluated the biomechanical behavior of four SIs (Figure 1d). Twelve compression (Figure 1e) tests are performed to determine which implant reduces *SS*. Finally, the results analysis indicates if the experiments were carried out correctly or if the technique used to obtain the specimens should be corrected (Figure 1f).

### 2.1. Device Description

**Figure 2:**
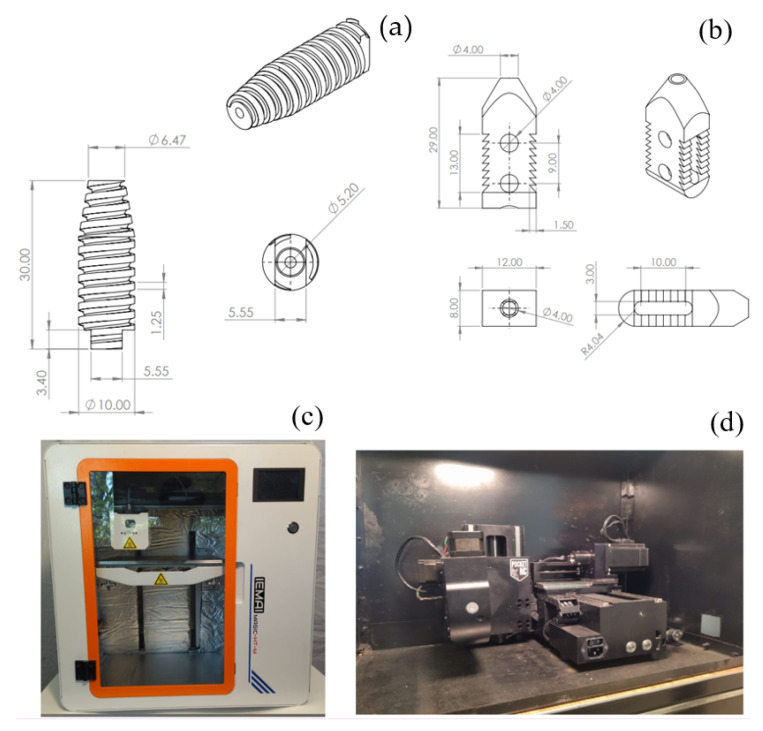
Spine implants diagram, dimensioning in mm. (**a**) Cannulated intersomatic screw with ACME thread for better fixation. (**b**) Titanium cage with eight teeth to better anchor. (**c**) MAGIC-HT-M machine. (**d**) Pocket NC V2-10 machine.

The screw (Figure 2a) is composed of 90% Titanium, 6% Aluminum, and 4% Vanadium. Where this alloy provides greater compatibility in the human body, the SI is fixed intersomatically between the lumbar spinous apophysis areas. It functions as a separator between two adjacent vertebrae for the rehabilitation of spinal disorders or fusion of intervertebral discs through a cannulated intersomatic screw, which has the particularity that the deep of the thread is fixed in the vertebral space to avoid any lateral slipping. The implant must have a diameter 2–5 mm larger than the prepared space for its colocation. It is introduced by opening its way and releasing the intervertebral tensions. At the same time, it generates an adequate fixation. It does not allow rotation or sliding movements after being removed from the positioning instrument, thus providing stability and immobility. This development can be used in mammals, preferably in humans. The cage’s structure is usually rectangular, as shown in Figure 2b. It is made of titanium, which is biocompatible and safe for use in the human body. The cage has holes or slots to allow surgeons to attach to the intervertebral disc securely. The cage interspinous spacer is surgically inserted between the spines to maintain an adequate distance between them, which helps restore the standard height of the spinal canal and reduce nerve compression. Symptoms such as pain and weakness in the extremities, which are often associated with spinal problems, are relieved. The eight teeth of the device help to anchor it. Rotation or displacement will not occur.

PEEK implants were printed with a MAGIC-HT-M machine (Dongguan Imai Intelligent Technology Co., Ltd., 2nd Floor, Building F, N0.6 Hupan road, Dalingshan Town, Dongguan, Guangdong, China) (Figure 2c). This product is specifically engineered for use with engineering plastics. It can withstand temperatures of up to 450 °C, with an extruder temperature of 150 °C, a hotbed temperature of 90 °C, and a chamber temperature of 90 °C. PEEK was printed at a speed of 15 mm/min. For the impression, the threads’ direction is parallel to the prosthesis longitudinal axis. Titanium prostheses were manufactured in Pocket NC V2-10 (Penta Machine Company, 119A Gold Miner Ln, Belgrade, MT 59714, USA) (Figure 2d). It can cut materials up to a hardness of G5 titanium, although it optimally cuts Delrin, aluminum, or softer steels. Travel is 128.3 mm (Y), 115.5mm (X), and 90.1mm (Z). Titanium is machined at 300 rpm and a feed rate of 6 mm per revolution.

### 2.2. Sample and Testing

Twelve porcine spinal segments, including eleven from the lumbar region and one from the thoracic region, were used for the experimental tests. A biomechanical porcine model was used because the bones of this animal have femoral dimensions, areas, lamellar bone structures, bone regeneration processes, bone mineral density, and concentration like humans [29]. With respect to vertebrae, the porcine lumbar spine can be used as an alternative animal model for the investigation of human lumbar spine biomechanics [30].

**Figure 3:**
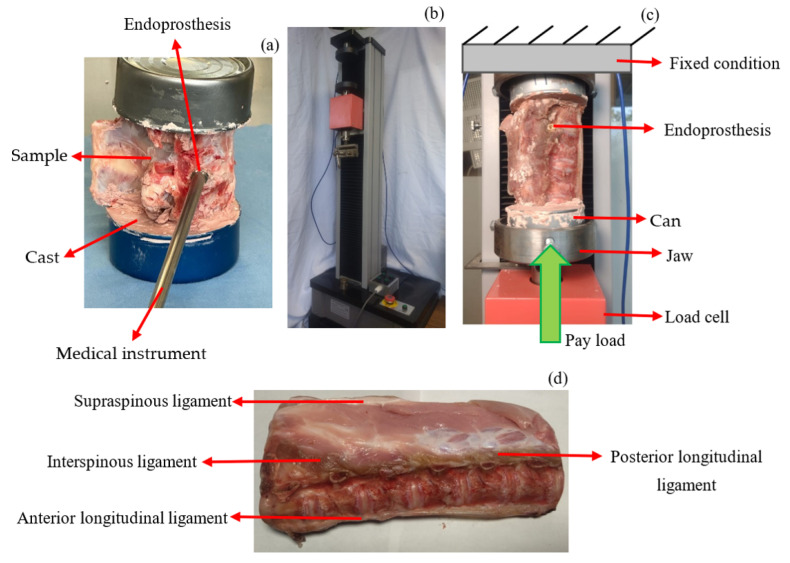
Scheme of the tests performed. (**a**) Endoprosthesis fit. (**b**) WDW-5 machine for compression tests. Load accuracy ≤ ±1%; displacement resolution is 0.01 mm. (**c**) Free body diagram. P is the load. (**d**) Anatomical description of the vertebrae.

Bones were obtained from twelve porcine corpses that were frozen at a temperature of minus 20 degrees Celsius, and muscle tissue was removed from vertebrae, with care to preserve spinal ligaments and facet joints. Before mechanical testing, the specimens were thawed at room temperature (20 degrees Celsius). Subsequently, a guide hole was drilled between the L3 and L4 vertebrae. The hole is 2 mm smaller than the diameter of the SI, so the tightening is sufficient and does not allow displacement of the prosthesis. The SI is placed with the help of the necessary medical instruments (Figure 3a). The lower and upper segments of the vertebrae are fixed with a dental cast in cans, which are placed in the jaws of WDW-5 Computer Control Electronic Universal Testing Machine (HST Group, Jinan Hensgrand Instrument Co., Ltd., 4915,West Jingshi Road, Jinan 250012, China), as shown in Figure 3b. ASTM E-9 [31] was used as a reference with the pertinent adjustments to perform the compression tests. The load was applied to the lower part of the vertebra (Figure 3c), and the upper part was fixed. Flesh and ligaments were removed from the bones in the first nine samples. The last three tests were performed with all the flesh and ligaments (Figure 3d). These included the anterior longitudinal ligament, posterior longitudinal ligament, interspinous ligament, and supraspinous ligament.

Twelve case studies were performed, and a compression load was applied to every single sample. The first four tests were done based on the load that supports a 50th percentile human; almost all the case studies were performed at a speed of 2 mm/min [32], except for sample 4, which was tested at a speed of 1 mm/min.

Table 1 summarizes the conditions applied to the study cases to perform the experimental tests.

Samples 1, 5, 8, 9, and 10 were measured until the system could not store more energy. Samples 2, 3, 4, and 12 were tested to 500 N because the lumbar region in normal standing posture supports 2/3 of the weight [33]. In the 50th percentile, it is approximately 500 N. Loads acting on the lumbar vertebrae during an activity such as walking can be as much as 2 to 2.5 times body weight. These weights are most significant at toe-off and increase as walking speed increases. In some activities like rowing, the lumbar region supports the body weight up to four times [34]. Considering the transmitted load to the lumbar region in physical activity (2.5–4 times body weight), a 50th percentile would experience a compressive force of 3000 N. Therefore, samples 6, 7, and 11 were tested until the load cell reached a value of 3000 N.

It is recommended to place SI into the sample and then fix it to the cans. MaxTest software (version 7.39, 2021, 4.20) was used to measure the machine’s jaw displacement and the load cell capacity. It is essential to specify that the software calculates the displacement of the load cell for all the tests performed, and due to the configuration of the experiment, the displacement of the load cell is equal to the deformation of the system.

### 2.3. Stress Shielding

It is proposed that the Hertz contact theory be used to quantify the stress between the SI and the hard tissue. According to [35] and with a modification, Equation (1) is useful to compute *SS*.

(1) $$SS=\sqrt{\frac{PE^{\prime }}{2\pi RL_{C}}}$$

where ^*E*′^ is the equivalent modulus of elasticity, L_C is the contact length, *P* is the applied load, and *R* is the SI’s radius. Equation (2) [35] shows how to compute ^*E*′^, and Equation (3) is proposed to evaluate the contact length between the SI and the cortical bone.

(2) $$E^{\prime }=\frac{2}{\frac{1-{\mu_{1}}^{2}}{E_{1}}+\frac{1-{\mu_{2}}^{2}}{E_{2}}}$$

(3) $$L_{C}=\frac{A_{C}}{V_{v}}$$

where $E_{1}$, $\mu_{1}$, $E_{2}$, and $\mu_{2}$ are the modulus of elasticity and Poisson’s ratio of the cortical bone and the SI, respectively, $V_{v}$ is the vertebrae volume, and $A_{C}$ is the contact area between the SI and the vertebrae. The equation is proposed because the researchers did not find an analytical method or model to compute the *SS*.

## 3. Results

**Figure 4:**
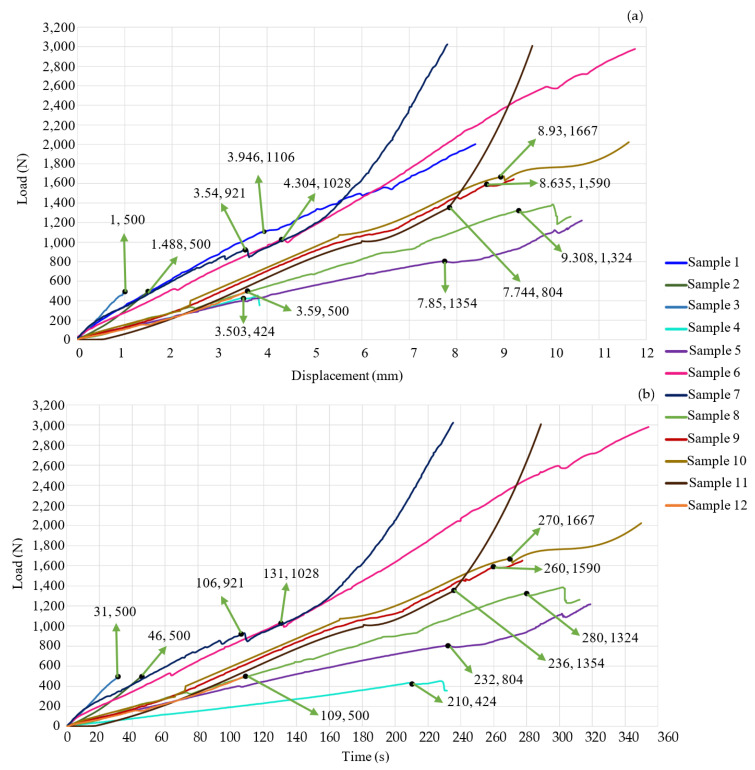
Experimental results. (**a**) Load displacement graph; (**b**) load-time graph. The indicated coordinates highlight the bone’s failure points.

**Figure 5:**
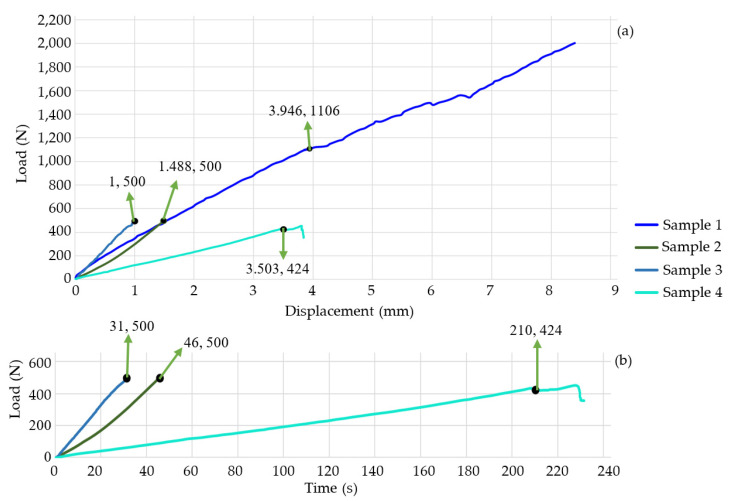
Experimental results. (**a**) Load-displacement graph; (**b**) load-time graph. The indicated coordinates highlight the failure points of the bone.

**Figure 6:**
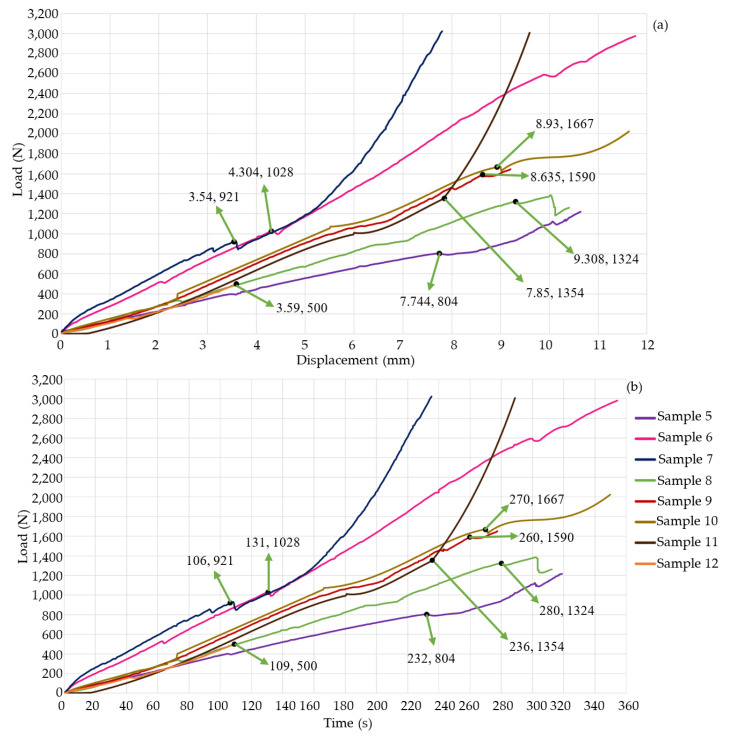
Experimental results. (**a**) Load-displacement graph; (**b**) load-time graph. The indicated coordinates highlight the failure points of the bone.

Figure 4a,b shows the results of the nine experiments. Figure 5a,b shows the experimental results of the first four study cases. Sample 1 failed when it supported a load of approximately 1.1 kN with a deformation of 3.946 mm. Despite the material’s failure, the graph indicates that the specimen continued storing energy; this is because titanium supports the increase in force. This means that the bone had no energy capacity. Therefore, in a patient, *SS* would occur, and the prosthesis would be embedded in the bone, which would generate pain, and the device would not fulfill its objective. The test was stopped at 252 s; test 2 ended at 46 s, and the machine reached a load of 496 N with a deformation of 1.49 mm. The behavior of the graph is linear because the material was not in the failure zone. The third specimen was preloaded with 500 N to apply an axial force of 500 N. Subsequently, the first preload caused a deformation of 1.49 mm, the test’s duration was 31 s, and it deformed 2.49 mm. The graph shows a linear behavior. Nevertheless, the material failed, and the bone lost its ability to store energy, so the deformation was faster than in the other three study cases. The time of the first and third tests were compared, and it was concluded that specimen 3 is 12.3% faster than test 1. The fourth specimen failed with a load of 424 N and a deformation of 3.5 mm. The test duration was 231 s. This test was performed on thoracic vertebrae. For that reason, the sample did not support a load of 500 N. Figure 6a,b show the last eight samples. Sample 5 reached a load of 804 N and a deformation of 7.744 mm. This experiment was performed without any SI, and the system would be able to store more energy, but after the critical load, the behavior was not linear; due to the experiment’s consideration, *SS* did not occur, and the test’s duration was 234 s. Sample 6 was performed by applying a maximum load of 3000 N, but the system failed with a compressive load of 1 kN and a deformation of 4.304 mm. It is possible to consider a linear behavior of the graph after the reported critical values, but Figure 6b shows variations in a short period. These fluctuations occur because the material loses the ability to store energy, and *SS* causes SI to be embedded in the bone. The test’s duration was 354 s. Sample 7 was tested until the load cell reached a value of 3000 N; the maximum load before the failure was 921 N, and the deformation was 3.54 mm. Due to *SS*, after the permissible load of the SI embedded in the bone, the experiment lasted 235 s.

Sample 8 lasted 312 s. The critical values were a load of 1324 N and a deformation of 9.308 mm. These experiments were performed with a PEEK cage. *SS* affects the entire system because the modulus of elasticity of PEKK (3.8 GPA) is smaller than that of cortical bone, and the critical load and deformation are bigger with PEEK than with metal. Comparing the ninth test (PEEK screw) against sample 7 (metal screw), Figure 6a,b prove that PEEK is a better option. So, *SS* is less in sample 9. The test finishes until the experiment reaches failure; the critical load is 1590 N, and the deformation is 8.635 mm. The test’s duration ends at 260 s. Tests 10, 11, and 12 were performed with PEKK screws. The mechanical behavior (slope) is similar among them. Test critical load was 1667 N and 1354 N with a deformation of 8.93 mm and 7.85 mm, for samples 10 and 11, respectively. Experiments ended when sample 10 reached the failure at 349 s and when sample 11 reached a 3000 N load at 289 s. In both experiments, *SS* occurred because the machine applied a bigger load than the system’s critical value. Test 12 was stopped when the load cell reached 500 N with a 3.59 mm deformation. The material did not fail. *SS* did not occur.

Figure 6a,b and Table 2 indicate that the metal SI produces a system’s critical deformation in the range of 3.5 to 4.5 mm, and there is a reduction in displacement (44.42–54.31%) with respect to the vertebrae’s deformation without any SI. In contrast, the PEEK prosthesis increases the deformation capability by 116.25% to 144% with respect to the metal ones. Also, with PEEK SI, the critical load increases in a range of 28.88% to 72.6%. Both increments (load and displacement) mainly depend on the sample’s geometrical properties. Sample 2 exhibited a linear behavior. It was about to fail due to its deformation in 46 s (too fast). This was mainly due to the dimensions of the sample. Based on the third test, it can be inferred that the *SS* causes the prosthesis to embed in the bone. The displacement reported on the graph is equivalent to how much the SI has become implanted in the bone. Test 4 supported 436 N before it failed. This is because the specimen was thoracic vertebrae, which have a lower stiffness than the lumbar vertebrae, keeping a lower load.

Figure 3a,b and Figure 5a,b show that the slope is directly proportional to the stiffness of the system. Sample 5 did not have any SI. Therefore, its longitudinal direction could deform more. Samples 1, 6, 7, 8, 9, 10, 11, and 12 exhibit a steeper slope than sample 5. This indicates an increase in the stiffness of every sample, but if the system is too rigid, it will fail with an impact or dynamic load. This can be verified with Figure 4 and Figure 5; the system was too stiff, and the critical load reached between 921 N and 1106 N, with a deformation of 3.5 mm to 4.3 mm. The stiffness increased because titanium alloy is harder than bone. *SS* will embed the metal prosthesis in the vertebrae. PEEK SI also has a steeper slope than sample 5, but it has a less steep slope than the metal SI. The PEEK samples exhibit the best biomechanical behavior. They reach a critical load from 1324 N to 1667 N. Compared to sample 5, The PEEK prosthesis increases the system’s displacement by 11.5% to 20.2%. The data indicates that *SS* will not cause the SI to embed, and the bone will not lose its capacity to support compressive loads. The comparison between the geometry of the cage and the screw remark that the cage distributes less energy than the screw. This is less useful for achieving the optimal biomechanical behavior. The results show that the best combination of geometry/material is the PEEK screw.

**Figure 7:**
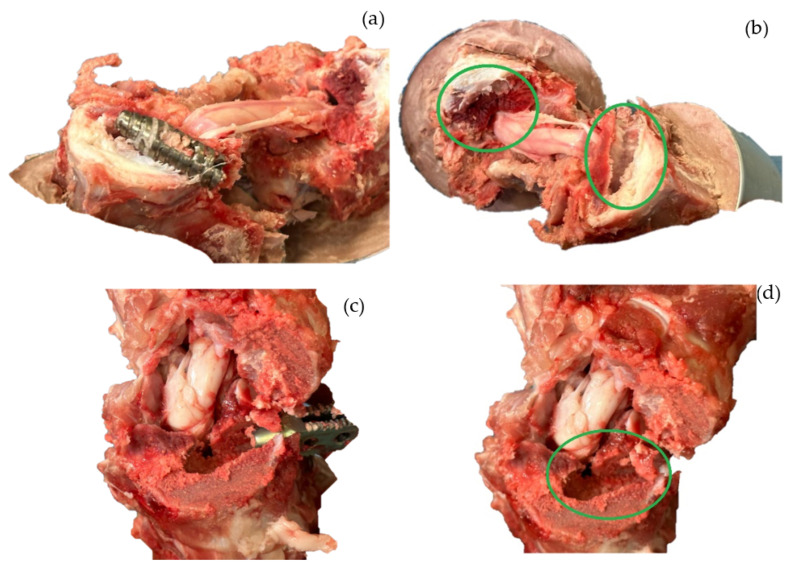
Vertebrae after mechanical testing. (**a**) Intersomatic screw (sample 3) without displacement in the bone. (**b**) Footprint left by the screw (sample 3) when it was embedded by stress shielding. (**c**) Cage (sample 4) without displacement in the bone. (**d**) Footprint left by the cage (sample 4) when it was embedded by stress shielding. The green circles indicate the marks caused by the Ti prostheses when embedded due to *SS*.

In tests 1, 3, and 4, the *SS* caused the prosthesis to embed into the bone when the vertebra was deformed by approximately 3.5 mm. The load at which this occurred varied according to the morphology and characteristics of the bone. Figure 7a,c show the position of the SI after the test. It can be seen that the prosthesis has not been displaced or rotated. Figure 7b,d show the mark that was generated when the bone lost load capacity and began to embed in the tissue. Figure 7b corresponds to sample 3, where the SI is implanted 1 mm between the vertebrae. Figure 7d corresponds to sample 4, where the SI is embedded at 0.34 mm. The mechanical behavior of the bone was due to *SS*.

**Figure 8:**
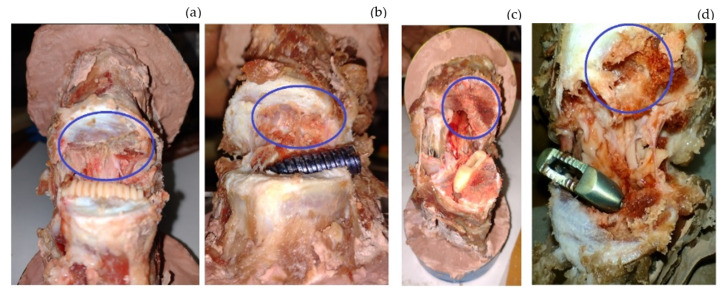
Vertebrae after mechanical testing, with blue circles highlighting the mark left by the SI. (**a**) Intersomatic screw (sample 9) footprint. (**b**) Intersomatic screw (sample 7) footprint: the screw was embedded by stress shielding. (**c**) Cage (sample 8) footprint. (**d**) Intersomatic screw (sample 6) footprint: the screw was embedded by stress shielding.

Figure 8a,b shows the intersomatic screw’s mark left by the *SS*. The metallic screw embeds more than the PEEK one. In fact, the PEEK screw only marked the tissue during the colocation; after that, there was no further displacement or embedding in the system. The metallic SI failed at a 3.54 mm deformation, and the test ended when the load cell reached 3000 N. Due to the *SS*, the metallic screw embedded 4.3 mm in the system. If this occurred in a patient, it would cause pain, and the SI would need to be removed. Furthermore, the comparison between the slopes of the screws shows that metal is stiffer than PEEK. The purpose of the SI is to create a simple fixation that maintains flexion, controls spinal motion, and maintains disc height to relieve pain. If the SI is too stiff, it will not achieve its objective, so the best material is PEEK.

Figure 8a,b also show the comparison between the materials used to manufacture the cages. Again, the PEEK one was not embedded in the cortical bone before the failure. Sample 6 had a displacement of 4.304 mm, and the test ended at an applied load of 3000 N. Due to the difference in stiffness between the materials and the *SS*, the prosthesis embedded 7.4567 mm. The comparison of the mark left by the metallic SI proves that the better geometry to design an SI is a screw form.

Table 3 shows the *SS* of the samples computed with Equations (1)–(3).

**Figure 9:**
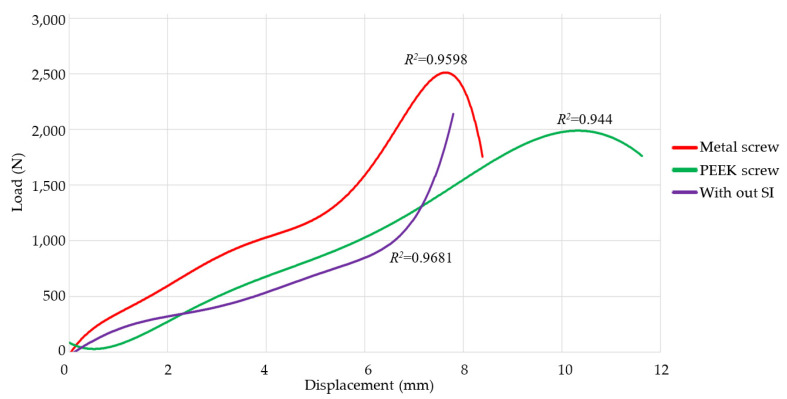
Polynomial regression of load-displacement plots of specimens 1, 2, 3, 7, 9, 10, 11, and 12.

Figure 9 illustrates the implementation of polynomial regression as a statistical technique to confirm the experimental findings and assess the consistency of the collected data. The selection of polynomial regression was determined by its capacity to represent intricate nonlinear relationships between independent and dependent variables, which is crucial when aiming to capture the exact dynamics of the experimental data. By applying polynomial regression to the collected data, one may assess the level of concordance among the values, thereby obtaining a quantitative assessment of the model’s precision.

Furthermore, polynomial regression facilitated the detection of patterns of variability in the data, thereby enabling the assessment of result repeatability. By comparing the polynomial curves generated for different data sets obtained under similar conditions, it was possible to confirm the consistency of the results, which is crucial to ensuring the credibility of the study’s conclusions. The strong connection between the replicated data and the polynomial model fit highlights the reliability of the experimental method and guarantees that the resulting findings can be replicated and reproduced. This methodology enhances the veracity of the findings, establishing a strong foundation for the interpretation of the results and the subsequent practical implementation of the conclusions.

Polynomial regressions were performed using the Ti and PEEK screw test data, as there were four tests conducted for each SI. The polynomial regression of the vertebra without an implant was computed using the data from sample 5 and the previously acquired experimental data from the biomechanics study group. Every case exhibits a regression exceeding 94%, which acts as a criterion for validating the replicability of the results. When comparing the functions associated with the SI, the PEEK (green) function is smoother than the Ti (red) function.

## 4. Discussion

*SS* of samples 1, 3, 4, 6, and 7 exceed the ultimate compressive strength of porcine cortical bone (68 MPa). Therefore, the SI is embedded into the cortical bone. According to Table 3, samples’ two *SS* is approximately 54 MPa; Figure 6a,b show there is not any variation in the slope because *SS* is smaller than hard tissue’s ultimate strength. The *SS* of samples 8, 9, 10, 11, and 12 prove that PEEK is better than titanium. The computed values do not exceed the maximum compressive strength of the bone. The SI would not be embedded in the system. The screw SI supports a more significant critical load than the cage. Also, the *SS* of the screw is smaller than the cage. The experiment’s limitations are that Vertebrae’s geometry causes the samples not to be aligned longitudinally with respect to the application of the load, and the eccentricity produces a bending moment that the software cannot measure. The sample consists of hard and soft tissue, metal cans, dental cast, and intervertebral discs. There are many materials, but the software only measures the applied load and the deformation of the entire system. Research does not consider how the SI interacts with all the forces (moments) generated by the body’s motion. If other loads were applied, the behavior in terms of the *SS* would be the same. PEEK would prove to be better than titanium. The only thing that could change would be the *SS* magnitude. The samples did not include a pedicle screw fixation system. Several researchers have reported the use of posterior internal fixation instrumentation to maintain stability at the operated level [36,37]. However, they did not evaluate the materials’ biomechanical behavior.

Shengjia et al. [38] and Yanfei et al. [39] performed a cage biomechanical evaluation with a finite element method. One of their study cases is the evaluation of the cage without a pedicle screw. They concluded that the pedicle screw fixations are better for reducing the stresses and the range of motions (ROM). The von Mises stresses and the ROM, when the implant is simulated alone, do not exceed the critical values for the material. Also, the fixation system reduces the parameters evaluated. However, they did not analyze *SS*. If the stresses are reduced, the bone will lose its ability to store energy and support load. If the 2 mm hole is smaller than the diameter of the SI, the stability is sufficient to ensure the correct operation of the implant. Moreover, the fixation system increases the *SS*, and eventually, this affects the bone.

During the test, the temperature and the speed at which the load is applied were monitored. The machine was calibrated according to ISO standards. Slopes’ tests 1, 2, 3, and 7 are similar to each other. The variations in results are not too significant. The same behavior occurred in tests 9, 10, 11, and 12. The results varied when they are totally different case studies, for example, 6 and 5. The criterion that validates the results is the comparison between similar case studies. Figure 7a,b show the effects of muscles and ligaments versus the clean samples. There is not a significant difference in biomechanical behavior. Tendons are fibrous structures that connect muscles to bones and allow joint mobility. When a muscle contracts, it generates tension in the tendon, which in turn causes the associated joint to move. The experiment was carried out with compression loads. The muscles and ligaments usually work in tension.

When comparing the biomechanical behavior reported in Figure 3, Figure 4 and Figure 5 with the results of Table 3, the equation proposed is in good agreement with the graphics’ failure points. When the critical load was reached in tests 1, 3, 4, 6, and 7, the computed *SS* was greater than the hard tissue’s ultimate strength. The SI started to embed into the cortical bone. Also, the graphics’ slope (Figure 3, Figure 4 and Figure 5) is not constant when the *SS* exceeds the ultimate strength. The change in the slope indicates material failure. The *SS* of test two agrees with the slope’s graphic. Since no critical value was exceeded, the SI was not embedded into the hard tissue. The graphics of tests 8, 9, 10, and 11 show a change in their slopes due to the system not being able to store more energy. Failure is imminent. The prosthesis did not embed because *SS* did not reach the hard tissue’s ultimate strength. The comparison between the slope and the proposed equation shows a good agreement between the computed values and the changes in the graphics when the system has failed. The biomechanical behavior of the nine tests indicates that PEEK exhibits a 63.53% *SS* reduction compared to the second test. In every scenario with respect to *SS*, the PEEK screw has the best performance.

SIs are designed with the objective of restoring foraminal height so that intervertebral pressure is reduced and pain is relieved. Due to their minimally invasive characteristics, SIs have become popular as a treatment for SDD, and there are various products on the market [40]. However, it is uncommon to perform experimental tests to determine the critical load and deformations at which *SS* occurs in the prosthesis. This study analyzes the biomechanical behavior of two SIs. The first is a new design of an intersomatic screw. The second is a cage. Both are manufactured from titanium and PEEK. Kangtai Ou et al. [41] indicated that SIs are significant in providing good mechanical stability to the surgical segment. This stability determines if the SI will have difficulties such as collapse or *SS*.

Figure 7b,d show that the prosthesis has become embedded in the bone. This is because there are different hardnesses between the materials. The stiffer one eventually penetrates the softer one, which can cause pain in the patient, preventing the SI from achieving its objective. The cage is less embedded than the screw. This is mainly because sample 3 had already been preloaded, and the test was completed until it supported a 500 N load. Test 4 (thoracic vertebrae) ended instantaneously after bone failure. Furthermore, the speed force was half that of the three previous experiments. Due to its geometry and the fact that it has fewer stress concentrators, the screw geometry is again the best option since it distributes the pressure in a better way throughout the system, allowing the bone to maintain a better load capacity and, therefore, reducing *SS*.

Bone remodeling is an ongoing process in which bone tissue is constantly rebuilt to adjust to mechanical demands, thus preserving bone strength and integrity. Nevertheless, the implementation of spinal implants can modify the natural distribution of loads, resulting in *SS*. This process occurs when the implant assumes most of the mechanical stress, thereby diminishing the stimulus for bone remodeling. Consequently, the bone may gradually become less strong, which could result in the failure of the implant or demand another surgery. PEEK implants are less stiff than titanium implants, which helps reduce *SS*. SI PEEK allows more stress to be transferred to the bone, promoting remodeling and reducing the risk of bone atrophy compared to SI Ti. As a result, PEEK implants may be more suitable for long-term outcomes, especially in preserving bone health.

Geometry is fundamental in SI. Zoubi et al. [42] and Singh et al. [43] reported that uniform and porous designs help to reduce *SS*, and this type of SI helps bone growth. The SI evaluated in this work exhibits similar behavior because it is a cannulated screw. It has the advantage of placing bone fragments in the segment to make a better connection between the two vertebrae and facilitate bone regeneration more effectively. With this similarity between the SI designs, it is inferred that the screw design helps to reduce *SS*. The thread is approximately 1 mm wide. The study [25] suggests that if the width is 0.5 mm, the stresses are transferred better throughout the system, which helps to reduce *SS*.

Nowadays, different SIs are designed with topological techniques [44,45], but these approaches focus on replicating the ranges of motion to resemble those of a healthy spine and reducing the pressure between the vertebrae and the discs. However, they do not analyze *SS*. Researchers who study the biomechanical behavior in prostheses [2,17,18,24,46,47,48] combine two techniques to reduce the *SS*: biomimetic porous manufacturing and topological approaches. An important point to highlight is that all the evaluations related to SIs [2,17,24] were done by FEM; the conclusions in the three studies indicate that the pores reduce the stiffness of the prosthesis, allowing the bone to maintain load capacity, which reduces *SS*. The loads applied in the mentioned studies differ from those involved in this work, but the biomechanical behavior of the prosthesis is similar. Due to the geometry of the SI, the bone maintains its load capacity, which reduces *SS*.

One aspect to consider in the future is to manufacture an SI with the same geometry and material, optimizing it with pores varying in the range of 0.4–0.6 mm and comparing its performance against the same design but manufactured from PEEK.

Future research could prioritize the enhancement of the shape and physical characteristics of SIs to further minimize the risk of *SS*. This may entail investigating bioactive coatings that stimulate bone formation and improve the osseointegration of the implant with the adjacent bone tissue. These coatings can mitigate the effects of *SS*. Furthermore, the inclusion of dynamic loading circumstances, such as those encountered during everyday activities, could offer a more comprehensive insight into the performance of implants in real-life scenarios. This has the potential to facilitate the creation of implants that are better equipped to accommodate diverse load conditions.

## 5. Conclusions

The factors to be considered for an optimal SI design and to reduce *SS* are the material, geometry, and porosity. The proposed design has the particularity that the deep teeth of the thread are anchored in the vertebral space to avoid any slippage. Having a transverse segment provides the possibility of placing bone remnants in part to make a better connection between the two vertebrae, achieving a better fixation of the screw. Additionally, being a cannulated screw, it has the advantage of placing the device in a precise way and without the risk of surgical injuries. The PEEK material proves to be better than titanium alloy. Moreover, the best SI is the screw because it supports more load before failure, has fewer stress concentrations, and has a better biomechanical behavior than a cage. These factors help to reduce *SS*. Finally, PEEK prostheses do not embed.

## Figures and Tables

**Table 1:** Study cases to perform compressive test analysis in porcine vertebrae L3–L4.

Study Case	Velocity Displacement (mm/min)	Prosthesis	Stop Criterion
1	2	Metal intersomatic screw.	Until material fail
2	2	Metal intersomatic screw.	Load of 500 N
3	2	Metal intersomatic screw.	Load of 500 N
4	1	Metal cage.	Load of 500 N
5	2	None	Until material fail
6	2	Metal cage.	Load of 3000 N
7	2	Metal intersomatic screw.	Load of 3000 N
8	2	PEEK cage.	Until material fail
9	2	PEEK intersomatic screw.	Until material fail
10	2	PEEK intersomatic screw.	Until material fail
11	2	PEEK intersomatic screw.	Load of 3000 N
12	2	PEEK intersomatic screw.	Load of 500 N

**Table 2:** Summary of the results of critical values obtained from the compressive tests.

Study Case	Critical Load (N)	Critical Displacement (mm)	Test’s Time (s)
1	1106	3.946	252
2	500	1.488	46
3	500	2.488	31
4	424	3.503	231
5	804	7.744	234
6	1028	4.304	354
7	921	3.54	235
8	1324	9.308	312
9	1590	8.635	260
10	1667	8.93	349
11	1354	7.85	289
12	500	3.59	109

**Table 3:** Summary of stress shielding achieved by the systems.

Study Case	Critical Load (N)	*SS* (MPa)
1	1106	80.2
2	500	53.92
3	500	76.26
4	424	71
6	1028	89.14
7	921	73.2
8	1324	36.93
9	1590	35.1
10	1667	35.93
11	1354	32.38
12	500	19.68

## Data Availability

The original contributions presented in the study are included in the article/Supplementary Material, further inquiries can be directed to the corresponding author.

## Supplementary Materials

The ASTM E-9 standard and several videos of the executed compression tests can be downloaded at https://www.mdpi.com/article/10.3390/jfb15080238/s1.
